# Cell proliferation and invasion ability of human choriocarcinoma cells lessened due to inhibition of Sox2 expression by microRNA-145

**DOI:** 10.3892/etm.2012.781

**Published:** 2012-10-30

**Authors:** FUHUI XU, HAIPENG WANG, XIA ZHANG, TE LIU, ZHIXUE LIU

**Affiliations:** School of Life Science and Technology, Tongji University, Shanghai 200092, P.R. China

**Keywords:** choriocarcinoma, microRNA-145, Sox2, proliferation, invasion

## Abstract

To date, the mechanism underlying the development of human choriocarcinomas has not been elucidated. It is hypothesized that the Sox2 protein plays a pivotal role in the proliferation and invasion capacity of tumor cells. A microRNA (miR-145) was cloned and used to study the expression of Sox2 and its regulatory effect on the proliferation and invasion capacity of the human choriocarcinoma cell line JAR. In the present study, *Sox2* mRNA and protein expression decreased in JAR and JEG-3 cells following transfection with the miR-145 expression virus. Cell proliferation assays indicated that miR-145 expression affected cell cycle regulation and suppressed the proliferation of choriocarcinoma cells *in vitro*. In addition, xenograft experiments confirmed the suppression of tumor growth *in vivo* due to cell cycle arrest. Therefore, endogenous mature miR-145 expression may have an important role in the pathogenesis of human choriocarcinomas via interference with the *Sox2* target gene by epigenetic modification. This information is of potential significance for the identification of therapeutic targets in human choriocarcinoma.

## Introduction

Human choriocarcinomas, which are a rare form of cancer that develops in the uterus from tissue that would normally become the placenta, are a trophoblastic gestational disease. These carcinomas have been studied largely to investigate conditions associated with pregnancy, such as preeclampsia (1). However, the mechanism underlying choriocarcinoma development remains to be elucidated.

microRNAs (miRNAs) are small RNA molecules (21–23 nt) that act as negative regulators of gene expression, either by blocking mRNA translation into protein or by RNA interference (2,3). Previous studies have revealed that dysregulation of specific miRNAs is associated with certain types of cancer and are considered to act either as oncogenes or tumor suppressors, depending on the target gene (2,4–10). Examples include, miR-15a, which has been associated with chronic lymphocytic leukemia (2,5,6), and also miR-21 and miR-17, which are upregulated, while miR-143 and miR-145 are down-regulated in colorectal cancer (11,12). The presence of the let-7 family of miRNAs is a prognostic factor in lung cancer (5,6) and miR-182 inhibits the proliferation and invasion ability of the human lung adenocarcinoma cells via its effect on human cortical actin-associated protein (13). To date, investigation of the decreased expression of miR-199b in human choriocarcinoma by Chao *et al* indicated that epigenetic mechanisms play an important role in increasing the expression levels of protein phosphatase 2A inhibitor and contribute to the pathogenesis of human choriocarcinoma (14). However, no studies have linked miR-145 expression with the proliferation and invasion capacity of human choriocarcinoma cells (2).

The transcription activator Sox2 was originally studied in the context of sexual determination during the development of *Drosophila* embryos and thus, its name is an acronym for ‘sex determination Y-box2’ (15–17). Numerous studies have indicated a primary role for Sox2 factor in the maintenance of embryonic stem cell pluripotency, and in later stages of development, in the repression of trophectoderm and epiblast genes. In addition, Sox2 appears to have a significant role in the differentiation of the nervous system (16). Extensive studies have indicated that Oct4, Sox2 and Nanog are required for self-renewal and pluripotency of embryonic stem cells (17,18). Investigation of the expression and methylation profiles of Sox2 in placentas and gestational trophoblastic disease by Li *et al* indicated that epigenetic mechanisms play an important role in the transcriptional regulation of Sox2 and contribute to the pathogenesis of gestational trophoblastic disease (19). By contrast, Xu *et al* reported that endogenous miR-145 represses the 3′-untranslated regions (3′-UTRs) of Oct4, Sox2 and Klf4, and that increased miR-145 expression inhibits human embryonic stem cell self-renewal, represses expression of pluripotency genes and induces lineage-restricted differentiation (18). In addition, Sox2 was closely associated with certain tumors, its inappropriate activation being involved in the development processes of human tumors, including the abnormal methylation modification of the promoter region of the Sox2 gene. Nakatsugawa *et al*(20) analyzed the functions of Sox2 in cancer stem-like cells/cancer-initiating cells derived from human lung adenocarcinoma. Nakatsugawa *et al* revealed that the Sox2 protein was detected in >80% of cancer stem-like cells/cancer-initiating cells in primary lung carcinoma tissues. However, Sox2 mRNA knockdown of the human lung cancer stem-like cells/cancer-initiating cells by gene-specific siRNA eliminated tumorigenicity *in vitro* and *in vivo*. These observations indicate that Sox2 has a role in the maintenance of stemness and tumorigenicity of human lung carcinoma and is a potential target for treatment.

In view of this evidence, in the current study, the miR-145 expression vector was transfected into the human choriocarcinoma cell lines JAR and JEG-3 to determine its specific role in Sox2 regulation and inhibition of cell proliferation, and invasion. These results are of potential importance for the identification of therapeutic targets in human choriocarcinoma.

## Materials and methods

### Cell lines and animals

The human choriocarcinoma cell lines JAR and JEG-3 were grown in DMEM (Hyclone, Logan, UT, USA) supplemented with 10% fetal bovine serum (PAA Laboratories Inc., Queensland, Australia), penicillin (100 U/ml), streptomycin (100 U/ml) and 2 mM L-glutamine (all were purchased from Hyclone). The JAR cells were incubated at 37°C in a humidified atmosphere of air containing 5% CO_2_. All experiments with BALB/c nude mice of 6–7 weeks of age and 20–22 g of weight were carried out at the Laboratory Animal Center of Tongji University with Institutional Animal Care and Use Committee approval in accordance with institutional guidelines.

### Recombinant lentivirus generation and vector construction

The method for creating the recombinant lentivirus package was as previously described (18). An RNAi pLL3.7 (LentiLox 3.7) lentiviral system was used to create lentiviral vectors (Clontech, Beijing, China). For vector pLL3.7-mir145 (pre-miRNA of miR-145 expression element), oligonucleotide pairs for pre-miRNA of miR-145 and linker sequences with *Hpa*I and *Xho*I sites were chemically synthesized. The sequences of the oligonucleotides were: top strand, 5′-CG**g tta ac**C ACC TTG TCC TCA CG**G TCC AGT TTT CCC AGG AAT CCC T**TA GAT GCT AAG ATG GGG ATT CCT GGA AAT ACT GTT CTT GAG GTC ATG GTT **ctc gag** CG-3′; and bottom strand, 5′-CG**c tcg ag**A ACC ATG ACC TCA AGA ACA GTA TTT CCA GGA ATC CCC ATC TTA GCA TCT AAG GGA TTC CTG GGA AAA CTG GAC CGT GAG GAC AAG GTG gtt aac CG-3′ (sequences corresponding to miR-145 seed sequences are capitalized and bold, and restriction enzyme sites are lower case and bold) (18). To build the expression plasmid, the pairs of oligos were annealed and inserted into the multiple cloning sites between the *Hpa*I and *Xho*I sites in the pLL3.7 vector. The negative control plasmid pLL3.7-mir145-Mut was similarly constructed, with the exception that 23 nucleotides in sequences corresponding to miR-145 were mutated (GTC CAG TTT TCC CAG GAA TCC CT to Gaa Ct Gaa TTa gCA cGA AgC aCT, mutations shown in lower-case). The pLL3.7-mir145 or pLL3.7-mir145-Mut was recombined in the package cell line 293T to create lentiviruses. Recombinant viruses were propagated in 293T cells, purified and titered by standard methods, as previously described by our laboratory (21). The corresponding viruses were designated Ldv-mir145 and Ldv-mir145-Mut. Co-transfection of human iPS cells used 4×10^7^ PFU/ml Ldv-mir145 or Ldv-mir145-Mut lentivirus according to the manufacturer’s instructions. The cells were seeded in a six-well plate in DMEM (Sigma-Aldrich, St. Louis, MO, USA) supplemented with 10% fetal bovine serum, 100 U/ml penicillin and 100 *μ*g/ml streptomycin at 37°C in a humidified atmosphere of air containing 5% CO*_2_*, until cells were 80% confluent.

### RNA extraction and analysis by quantitative real-time PCR (qRT-PCR)

Total RNA from each cell was isolated using TRIzol reagent (Invitrogen, Carlsbad, CA, USA) according to the manufacturer’s instructions. The RNA samples were treated with DNase I (Sigma-Aldrich), quantified and reverse-transcribed into cDNA using the ReverTra Ace-α First Strand cDNA Synthesis kit (Toyobo, Osaka, Japan). qRT-PCR was conducted using a RealPlex4 real-time PCR detection system from Eppendorf Co., Ltd., (Hamburg, Germany), with SYBR Green RealTime PCR Master mix (Toyobo) used as the detection dye. qRT-PCR amplification was performed over 40 cycles with denaturation at 95°C for 15 sec and annealing at 58°C for 45 sec. Target cDNA was quantified using the relative quantification method. A comparative threshold cycle (Ct) was used to determine gene expression relative to a control (calibrator) and steady-state mRNA levels were reported as an n-fold difference relative to the calibrator. For each sample, the marker genes Ct values were normalized using the formula ΔCt=Ct_genes-Ct_18S rRNA. To determine relative expression levels, the following formula was used: ΔΔCt= ΔCt_all_groups-ΔCt_blankcontrol_group. The values used to plot relative expressions of markers were calculated using the expression 2^−ΔΔCt^. The mRNA levels were calibrated based on levels of 18S rRNA. The cDNA of each gene was amplified using primers as previously described (19).

### Methyl-thiazolyl-tetrazolium (MTT) assay for cell proliferation

Each group of JAR and JEG-3 cell lines was seeded at 2×10^3^ per well in 96-well plates and cultured in DMEM supplemented with 10% FBS at 37°C with 5% CO_2_, until cells were 85% confluent. The MTT (Sigma Chemicals, St. Louis, MO, USA) reagent (5 mg/ml) was added to the maintenance cell medium at various time points and incubated at 37°C for an additional 4 h. The reaction was terminated with 150 *μ*l dimethylsulfoxide (DMSO, Sigma Chemicals) per well and the cells were lysed for 15 min, and the plates were agitated every 5 min. Absorbance values were determined using the enzyme linked immunosorbent assay (ELISA) reader (Model 680; Bio-Rad, Hercules, CA, USA) at 490 nm.

### Flow cytometric (FCM) analysis of cell cycle by propidium iodide (PI) staining

Each group of JAR and JEG-3 cell lines was seeded at 3×10^5^ per well in 6-well plates and cultured until 85% confluent. Each group of cells was washed with PBS three times, then collected by centrifuging (Allegra X-22R; Beckman Coulter, Miami, FL, USA) at 1000 × g for 5 min. The cell pellets were resuspended in 1 ml PBS, fixed in 70% ice-cold ethanol and kept in a freezer for >48 h. Prior to FCM analysis, the fixed cells were centrifuged, washed twice with PBS and resuspended in PI staining solution (Sigma Chemicals) containing 50 *μ*l/ml PI and 250 *μ*g/ml RNase A (Sigma Chemicals). The cell suspension, which was kept in the dark, was incubated for 30 min at 4°C and analyzed by FACS (FCM-500, Beckman Coulter). A total of 20,000 events were acquired for analysis using CellQuest software.

### Luciferase report assay

All steps of the luciferase reporter assay were as previously described (18,22,23). NIH-3T3 cells were seeded at 3×10^4^ per well in 48-well plates and co-transfected with 400 ng pLL3.7-mir145, pLL3.7 or pLL3.7-mir145-Mut, 20 ng pGL3cm-Sox2-3UTR-WT or pGL3cm-Sox2-3UTR-Mut, and pRL-TK (Promega, Madison, WI, USA) using Lipofectamine 2000 reagent according to the manufacturer’s instructions. Luciferase activity was measured 48 h after transfection using the dual-luciferase reporter assay system (Promega).

### RNA extraction and northern blot analysis

Northern blotting was performed as previously described (13,24). For all groups, 20 *μ*g good quality total RNA was analyzed on a 7.5 M urea 12% PAA denaturing gel and transferred to a Hybond N^+^ nylon membrane (Amersham, Freiburg, Germany). Membranes were crosslinked using UV light for 30 sec at 1,200 mJ/cm^2^. Hybridization was performed with the miR-145 antisense starfire probe, 5′-AGG GAT TCC TGG GAA AAC TGG AC-3′ (IDT, Coralville, IA, USA), to detect the 22-nt miR-199a fragments according to the manufacturer’s instructions. After washing, membranes were exposed for 20–40 h to Kodak XAR-5 films (Sigma-Aldrich). As a positive control, all membranes were hybridized with a human U6 snRNA probe, 5′-GCA GGG GCC ATG CTA ATC TTC TCT GTA TCG-3′. Exposure times for the U6 control probe varied between 15 and 30 min.

### Western blot analysis

Total protein extracts from each group of cells were resolved by 12% SDS-PAGE and transferred on PVDF (Millipore, Billerica, MA, USA) membranes. After blocking, the PVDF membranes were washed 4 times for 15 min with TBST at room temperature and incubated with the primary antibody [rabbit anti-human Sox2 polyclonal antibody (1:200; Chemicon, Temecula, CA, USA)]. After extensive washing, membranes were incubated with secondary peroxidase-linked goat anti-rabbit IgG (1:1000; Santa Cruz Biotechnology, Inc., Santa Cruz, CA, USA) for 1 h. After washing 4 times for 15 min with TBST at room temperature, the immunoreactivity was visualized by enhanced chemiluminescence (ECL kit; Pierce Biotechnology, Inc., Rockford, IL, USA) and the membranes were exposed to Kodak XAR-5 films.

### Soft agar colony formation assay

The method used was as previously described (25). Soft agar assays were constructed in 6-well plates. The base layer of each well consisted of 2 ml with final concentrations of 1X medium (DMEM+10% FBS) and 0.6% low melting point agarose. The plates were chilled at 4°C until solid. Subsequently, a 1.0-ml agar growth layer, consisting of 1×10^4^ cells suspended in 1X media and 0.3% low melting point agarose, was poured onto the base layer. The plates were again chilled at 4°C until the growth layer congealed. Additional 1X media without agarose (1.0 ml) was added to the top of the growth layer on day 0 and again on day 15 of growth. The cells were allowed to grow at 37°C for 1 month and total colonies counted. The assays were repeated a total of 3 times. Results were statistically analyzed by paired t-test using the PRISM Graphpad program (Graphpad Software, La Jolla, CA, USA).

### Transwell migration assay

All steps were as previously described (26). The cells (2×10^5^) were resuspended in 200 *μ*l serum-free medium and seeded on the top chamber of the 6.5 mm polycarbonate transwell filters (8.0 *μ*m pores; Corning Inc., Corning, NY, USA). The full medium (600 *μ*l) containing 10% FBS was added to the bottom chamber. The cells were allowed to migrate for 24 h at 37°C in a humidified incubator with 5% CO_2_. The cells attached to the lower surface of the membrane were fixed in 4% paraformaldehyde at room temperature for 30 min and stained with 4,6-diamidino-2-phenylindole (DAPI; C1002; Beyotime Institute of Biotechnology, Jiangsu, China), and the number of cells on the lower surface of the filters was counted under the microscope. A total of 5 fields were counted for each transwell filter.

### In vivo xenograft experiments

Logarithmically growing ovarian cancer-initiating cells (∼1×10^5^) were inoculated into BALB/c nude/nude mice. Each experimental group consisted of four mice. After 4 weeks of observation, the mice were sacrificed and tumors were obtained (27). The tumor weight was measured and tumor volume was calculated according to the formula: tumor volume (mm^3^) = length (mm) × width (mm) × height (mm).

### Statistical analysis

Each experiment was performed at least three times and data were expressed as the mean±SE. The differences were evaluated using Student’s t-tests. P<0.05 was considered to indicate a statistically significant result.

## Results

### miR-145 binding with the 3′-UTR sites in Sox2

**Figure 1:**
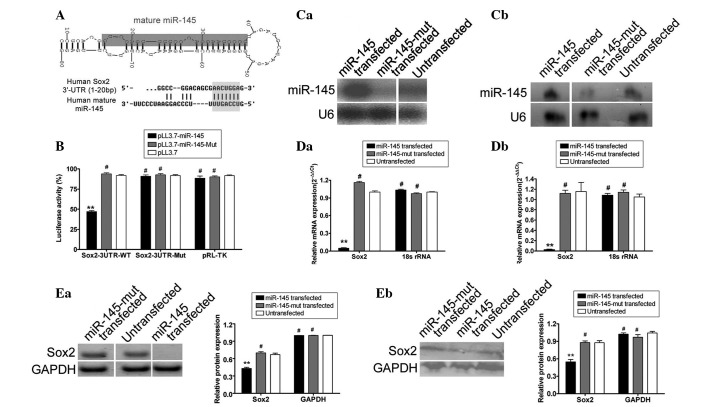
miR-145 and Sox2 expression in different groups. (A) The human *Sox2* microRNA (miRNA) 3′-untranslated region (3′-UTR) contains miR-145 binding sites. The mature miR-145 sequences of multiple species were analyzed and contrasted using bioinformatics tools. The typical secondary structure of precursor miRNAs (pre-miRNAs) was compared with miR-145. Pre-miRNA contains stem-loop and hairpin structures and the common binding site is located in an unstable region with a multi-branching loop-like RNA structure. Mature miRNAs are bound in the 3′-UTR of the target gene. Complementarity between miR-145 and the putative human *Sox2* 3′-UTR site target (1–20 bp downstream) showed that the conserved bases of the putative miR-145 target sequence are present in the human *Sox2* 3′-UTR. (B) The expression of miR-145 and its interference with the target gene *Sox2* were assessed by luciferase assays. Wild-type (wt) reporter or mutated control luciferase plasmids were transfected into NIH-3T3 cells with miR-145 or mutant miR-145 expression viruses. Luciferase activity within the *Sox2* 3′-UTR sites was inhibited by miR-145 (^**^P<0.01 vs. pLL3.7; #P>0.05 vs. pLL3.7; n=3). (C) Northern blot hybridized signals of miR-145 in human choriocarcinoma cells. Northern blot hybridized signals of miR-145 in (Ca) JAR cells and (Cb) JEG-3 cells. Northern blot analysis showed a weaker hybridized signal in mutant miR-145-transfected cells than in wt miR-145-transfected cells. The human U6 probe was used as a loading control. (D) *Sox2* mRNA expression assay in different human choriocarcinoma cells by quantitative real-time PCR (qRT-PCR). qRT-PCR indicated lower expression of *Sox2* mRNA in the (Da) wt miR-145-transfected JAR cells and (Db) wt miR-145-transfected JEG-3 cells than in the corresponding untransfected or mutant miR-145-transfected cells. Relative mRNA expression is shown after normalization to 18S rRNA, which served as an internal control. (E) Western blot showing the expression of Sox2 in various groups. Western blots showing the expression of Sox2 in (Ea) JAR cells and (Eb) JEG-3 cells. Sox2 levels were significantly higher in the untransfected or mutant miR-145-transfected cells than in the miR-145-transfected cells. Data indicate that exogenous miR-145 downregulates Sox2 expression (^**^P<0.01 vs. untransfected; ^#^P>0.05 vs. untransfected; n=3).

Using an online research tool, the miRBase Target database (http://www.mirbase.org) (28,29), the precursor miRNA (pre-miRNA) sequences, mature miRNA sequences, chromosomal locations and length of miR-145 and the target gene *Sox2* were analyzed. Seven putative miRNA target sites were identified in the 3′-UTR of *Sox2* mRNA, depending on species. This study focused on human miR-145, which targets the human *Sox2* 3′-UTR, although conservation in this sequence indicates the possibility of binding to varying degrees, across species (Fig. 1). Plasmid DNA encoding each *Sox2* mRNA 3′-UTR site [wild-type (wt) *Sox2*, empty plasmid and mutant *Sox2*] was co-transfected with the miR-145 expression lentivirus (wt miR-145, empty lentivirus and mutant miR-145 lentivirus) into the mouse embryonic fibroblast cell line NIH-3T3, to examine regulation of *Sox2* gene expression by mature miR-145. The luciferase activity of the *Sox2* 3′-UTR sites was significantly inhibited by wt miR-145 (Fig. 1), while the luciferase activity of the mutated *Sox2* 3′-UTR sites was not inhibited, suggesting that *Sox2* was targeted by miR-145.

### miR-145 specifically influences expression of Sox2 protein in human choriocarcinoma cell lines

Northern blot analysis demonstrated that the hybridized signal of mutant miR-145 in the JAR and JEG-3 choriocarcinoma cell lines was weaker than in cells transfected with wt miR-145. qRT-PCR and western blot analyses were used to determine the effect of exogenous and endogenous miR-145 expression on Sox2 expression. qRT-PCR analyses revealed decreased *Sox2* mRNA expression in wt miR-145 lentivirus-transfected JAR and JEG-3 cells than in untransfected and mutant miR-145-transfected cells. The relative mRNA expression after normalization to 18S ribosomal RNA (rRNA), which served as an internal control, is shown in Fig. 1. Notably, western blotting revealed that Sox2 levels in untransfected cells (JAR or JEG-3 cell lines) and mutant miR-145 transfected cells (JAR or JEG-3 cell lines) were 0.667±0.026 or 0.876±0.036, and 0.669±0.020 or 0.879±0.028 relative to those of GAPDH, respectively (Fig. 1). These values were significantly higher than those for the wt miR-145 transfected group (JAR: 0.429±0.019; JEG-3: 0.547±0.040 relative to GAPDH), which indicated that exogenous miR-145 down-regulated Sox2 expression. Therefore, miR-145 expression may influence endogenous Sox2 expression.

### Proliferation and invasion of human choriocarcinoma cell lines were inhibited by miR-145

**Figure 2:**
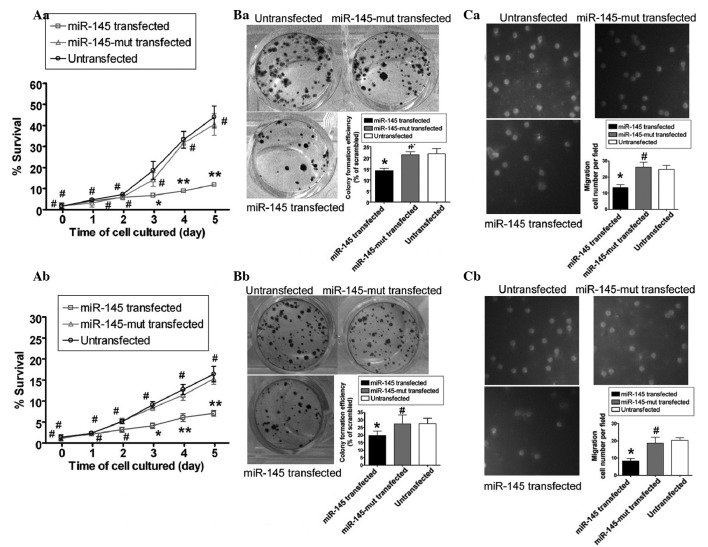
Exogenous miR-145 influences the proliferation and invasion capacity of the human choriocarcinoma cells. (A) Methyl-thiazolyl-tetrazolium (MTT) assays showing the survival rate in various cells. The survival rates of miR-145-transfected (Aa) JAR cells and (Ab) JEG-3 cells were markedly lower than those of the corresponding untransfected and mutant miR-145 transfected cells, at 3 and 5 days post-transfection. (^*^P<0.05 vs. untransfected cells; ^**^P<0.01 vs. untransfected cells; ^#^P>0.05 vs. untransfected cells; n=3). (B) The results of the transwell migration invasion assay. The numbers of invading cells were significantly lower for miR-145-transfected (Ba) JAR cells and (Bb) JEG-3 cells than for the corresponding untransfected and mutant miR-145-transfected cells. (^*^P<0.05 vs. untransfected cells; ^#^P>0.05 vs. untransfected cells; n=3). (C) The results of soft agar colony formation assay. Soft agar colony formation assays consistently indicated that miR-145-transfected (Ca) JAR cells and (Cb) JEG-3 cells formed substantially fewer colonies than the corresponding control or mutant miR-145-transfected-cells, when plated at low density. (^*^P<0.05 vs. untransfected cells; ^#^P>0.05 vs. untransfected cells; n=3). These results suggest that miR-145-mediated repression of Sox2 expression significantly attenuates the invasion and migration capacity of human choriocarcinoma cells.

**Figure 3:**
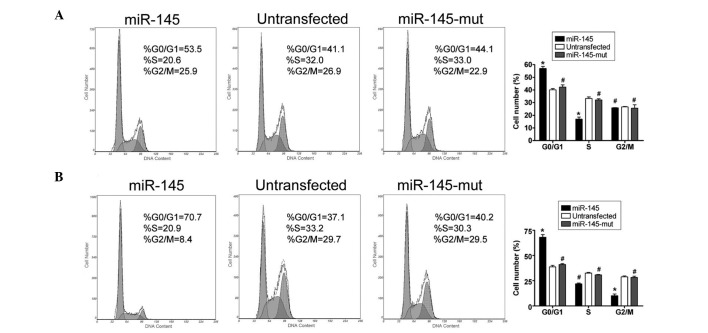
Exogenous miR-145 influences the cell cycle in the human choriocarcinoma cells. (A) Flow cytometric analysis showed that the majority of the miR-145-transfected JAR cells were arrested in the G_0_/G_1_ phase of the cell cycle and the percentage of cells in the S phase was markedly decreased. (B) Flow cytometric analysis showed that the majority of the miR-145-transfected JEG-3 cells were arrested in the G_0_/G_1_ phase of the cell cycle and the percentage of cells in the G_2_/M phases was markedly decreased. (^*^P<0.05 vs. untransfected cells; #P>0.05 vs. untransfected cells; n=3).

The results of the proliferation assays performed are shown in Fig. 2. Using an MTT assay, the survival rate of wt miR-145 lentivirus-transfected cells was demonstrated to be markedly lower than that of untransfected cells and mutant miR-145-transfected cells at both 3 and 5 days post-transfection. By contrast, no differences in viability were observed in untransfected cells, mutant miR-145-transfected cells and wt miR-145 transfected cells 1 and 2 days post-transfection. The survival rates remained unchanged for untransfected cells and mutant miR-145 transfected cells for the remainder of the time course, which indicated that induced exogenous miR-145 expression inhibited the growth of human choriocarcinoma JAR and JEG-3 cell lines *in vitro*. In addition, migration and invasion ability were shown to be reduced in JAR and JEG-3 cells with stably repressed Sox2 mediated by miR-145 transfection using transwell migration analysis and soft agar colony formation assays, respectively (Fig. 2). Transwell migration invasion assays showed that the number of invading wt miR-145-transfected JAR cells was significantly lower than the numbers of invading untransfected and mutant miR-145-transfected JAR cells (invading cell numbers: miR-145 transfected group, 13±2; untransfected cells, 24±2; mutant miR-145 transfected cells, 26±2). The results of transwell migration invasion assays in JEG-3 cells were similar to those of JAR cells. These results indicate that the repression of Sox2 expression by miR-145 significantly attenuates the invasion and migration ability of human choriocarcinoma cells. Soft agar colony formation assays consistently indicated that miR-145-transfected cells formed substantially fewer colonies compared with controls or mutant miR-145-transfected cells when plated at low density (Fig. 2). In addition, miR-145 transfected, mutant miR-145-transfected and untransfected JAR or JEG-3 cells were stained with PI, and analyzed by flow cytometry to detect changes in cell cycle progression. As shown in Fig. 3, the majority of the wt miR-145-transfected JAR cells were arrested in the G_0_/G_1_ phase of the cell cycle and the percentage of cells in the S phase were markedly decreased. By contrast, no significant differences were observed in the cell cycle distribution of the mutant miR-145-transfected and untransfected JAR cells. In addition, the majority of wt miR-145-transfected JEG-3 cells were arrested in the G_0_/G_1_ phase of the cell cycle and the percentage of cells in the G_2_/M phase were markedly decreased. However, no significant differences were observed in the cell cycle distribution of the mutant miR-145-transfected and untransfected cells. The results suggested that wt miR-145 expression affected cell cycle regulation in human choriocarcinoma cells *in vitro*.

### Expression of wt miR-145 in JAR cells inhibited subcutaneous tumor growth in nude mice

**Figure 4:**
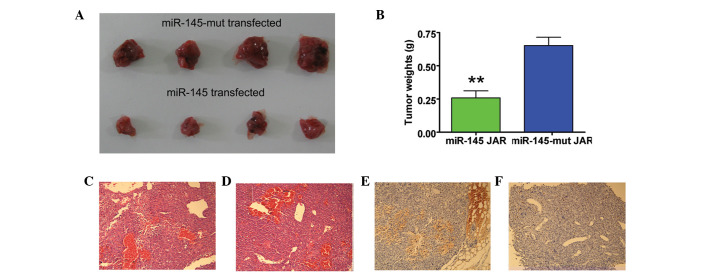
Xenograft experiment results. (A) All mice in the mutant miR-145-transfected group developed tumors ∼37 days after injection, while tumors formed in the miR-145-transfected group grew more slowly. (B) Tumor weights in the miR-145-mut group were heavier than in the miR-145-transfected group, and miR-145 expression was associated with decreased the tumor volume (^**^P<0.01 vs. nontransfected; n=4). Hematoxylin and eosin staining revealed cellular heterogeneity in pathological sections of excised tumor tissue from (C) the mutant miR-145-transfected group and (D) the miR-145-transfected group (original magnification: ×200). Immunohistochemistry showing (E) positive or strongly positive Ki-67 staining in tumors formed by mutant miR-145-transfected JAR cells and (F) weakly positive Ki-67 staining in miR-145-transfected cells. Cell proliferation-related protein Ki-67 was a biomarker to evaluate the balance of tumor cell proliferation and programmed cell death (original magnification: ×200).

The effect of miR-145 expression on tumor growth was investigated *in vivo* by subcutaneous inoculation of the miR-145 lentivirus-transfected JAR cells and mutant miR-145 lentivirus-transfected JAR cells into two groups of nude mice. All the mice in the mutant miR-145 group developed tumors ∼37 days after injection, whereas tumors were detected in only one in four mice from the miR-145 transfected group at this time. Although both groups developed tumors, the tumors formed by wt miR-145-transfected cells grew more slowly than those in the mutant miR-145-transfected group (Fig. 4). In addition, when the mice were sacrificed 62 days after injection, tumor weights in the mutant miR-145 transfected group were significantly heavier than those in the wt miR-145 lentivirus-transfected group. Furthermore, miR-145 expression in the JAR cell line was associated with a significant decrease in tumor volume (Fig. 4). These results suggested that miR-145 expression in the human choriocarcinoma cell line suppressed *in vivo* tumor growth.

## Discussion

Increasing evidence has shown that miRNA plays an important role in the proliferation and invasion ability of numerous types of cancer cells. However, the miRNAs that regulate human choriocarcinoma cell growth and invasion have not yet been reported. In the present study, it was observed that miR-145 interfered with Sox2 expression via putative sites located in the 3′-UTR region. Therefore, it was hypothesized that miR-145 suppresses the human choriocarcinoma cell line JAR by downregulation of Sox2 expression. Putative miRNA target sites in the 3′-UTR of *Sox2* mRNA were used to construct a wt miR-145 expression lentivirus, which was then transfected into the JAR human choriocarcinoma cell line. Luciferase activity assays indicated that the activity of the *Sox2* 3′-UTR site was significantly inhibited by wt miR-145, while that of the mutated *Sox2* 3′-UTR site was unchanged, which suggested that miR-145 targeted *Sox2*. In addition, qRT-PCR and western blot analysis demonstrated that Sox2 protein expression was reduced in wt miR-145 lentivirus-transfected JAR cells compared with the levels detected in mutant miR-145 lentivirus-transfected or untransfected cells. FCM analysis revealed that the majority of wt miR-145-transfected cells were arrested in the G_0_/G_1_ phase of the cell cycle with reduced percentages in the S and G_2_/M phases, which suggested that miR-145 expression affected the cell cycle regulation of choriocarcinoma cells *in vitro*. Similarly, exogenous miR-145 expression was shown to inhibit the growth of the JAR cell line *in vitro* using MTT assays. Soft agar colony formation assay and transwell migration invasion assays showed that the number of invading wt miR-145 transfected cells was significantly lower than the numbers of invading untransfected and mutant miR-145 transfected cells. Finally, xenograft experiments indicated that miR-145 expressed in the JAR cell line also suppressed tumor growth *in vivo*.

This is in contrast to a previous report which found that the expression of high levels of Sox2 was associated with malignancy in human lung cancer stem-like cells/cancer-initiating cells (20). In studies of human choriocarcinoma, it was observed that expressed levels of Sox2 in human gestational trophoblastic neoplasia cells were higher than in the normal trophoblast cells (19). Thus, we considered that there was an association between Sox2 expression and malignancy in human choriocarcinoma. By contrast, certain studies have also shown that miR-145 may specifically regulate the target gene Sox2 expression (21). Therefore, we investigated whether the expression of endogenous Sox2 in human choriocarcinoma was silenced when the miR-145 overexpression might weaken the proliferation and invasion of carcinoma. In the current study, exogenous miR-145 was transfected into the two human choriocarcinoma cell lines, JAR and JEG-3 using a lentiviral system. The effectiveness of miR-145 overexpression not only as an inhibitor of endogenous Sox2 expression, but also as a suppressor of proliferation and invasion in human choriocarcinoma cell lines, was investigated in several ways, including cell proliferation, invasion and infiltration assays, and tumorigenicity assays in nude mice. The results suggest that not only proliferation, but also invasion and infiltration were reduced following miR-145 overexpression in the human choriocarcinoma cell lines, JAR or JEG-3. In comparison with the wt cell lines, the tumorigenicity in nude mice of JAR cells transfected with exogenous miR-145 was reduced. These results suggest that the proliferation and invasion capacity of human choriocarcinoma cells is associated with Sox2 expression. It may be speculated that inhibition or loss of miR-145 expression results in excessive Sox2 expression, and therefore influences tumor growth. Further studies are required to fully elucidate the function of miR-145 in this process. However, it is clear that miR-145 and Sox2 play potentially important roles in the pathogenesis of human choriocarcinomas.
